# (-)- Gossypol Inhibition of Musashi-Mediated Forgetting Improves Memory and Age-Dependent Memory Decline in *Caenorhabditis elegans*

**DOI:** 10.1007/s12035-022-03116-7

**Published:** 2022-11-15

**Authors:** Pavlina Mastrandreas, Andreas Arnold, Csaba Boglari, Dominique J.-F. de Quervain, Attila Stetak, Andreas Papassotiropoulos

**Affiliations:** 1 Transfaculty Research Platform Molecular and Cognitive Neurosciences, University of Basel, Birmannsgasse 8, 4055 Basel, Switzerland; 2 Division of Molecular Neuroscience, Department of Psychology, University of Basel, Birmannsgasse 8, 4055 Basel, Switzerland; 3 Biozentrum, Life Sciences Training Facility, University of Basel, Klingelbergstrasse 50/70, 4056 Basel, Switzerland; 4 Division of Cognitive Neuroscience, Department of Psychology, University of Basel, Birmannsgasse 8, 4055 Basel, Switzerland; 5 University Psychiatric Clinics, University of Basel, Wilhelm Klein-Strasse 27, 4055 Basel, Switzerland

**Keywords:** Musashi, Forgetting, (-)- Gossypol, *C. elegans*, Ageing

## Abstract

Musashi RNA-binding proteins (MSIs) retain a pivotal role in stem cell maintenance, tumorigenesis, and nervous system development. Recently, we showed in *C. elegans* that Musashi (MSI-1) actively promotes forgetting upon associative learning via a 3’UTR-dependent translational expression of the Arp2/3 actin branching complex. Here, we investigated the evolutionary conserved role of MSI proteins and the effect of their pharmacological inhibition on memory. Expression of human Musashi 1 (MSI1) and Musashi 2 (MSI2) under the endogenous Musashi promoter fully rescued the phenotype of *msi-1(lf)* worms. Furthermore, pharmacological inhibition of human MSI1 and MSI2 activity using (-)- gossypol resulted in improved memory retention, without causing locomotor, chemotactic, or learning deficits. No drug effect was observed in *msi-1(lf)* treated worms. Using Western blotting and confocal microscopy, we found no changes in MSI-1 protein abundance following (-)- gossypol treatment, suggesting that Musashi gene expression remains unaltered and that the compound exerts its inhibitory effect post-translationally. Additionally, (-)- gossypol suppressed the previously seen rescue of the *msi-1(lf)* phenotype in worms expressing human MSI1 specifically in the AVA neuron, indicating that (-)- gossypol can regulate the Musashi pathway in a memory-related neuronal circuit in worms. Finally, treating aged worms with (-)- gossypol reversed physiological age-dependent memory decline. Taken together, our findings indicate that pharmacological inhibition of Musashi might represent a promising approach for memory modulation.

## Introduction

Learning and memory constitute cognitive functions comprising a wide variety of complex components. Even though there has been a lot of effort to elucidate the molecular aspects of memory [1–3], actively controlled forgetting is an equally important but much less investigated mechanism [4–6]. We have recently demonstrated in *C. elegans* that the sole Musashi RNA-binding protein (RBP) plays an important regulatory role in forgetting by modulating cytoskeletal changes at the translational level [7]. The Musashi protein was originally discovered as a key player in asymmetric cell division, stem cell function, and cell fate determination in *Drosophila* [8]. In vertebrates, the two *MSI* orthologues, *Musashi1* (*MSI1*) and *Musashi2* (*MSI2*), are mostly expressed in stem cells. In particular, MSI1 is a marker of neural progenitor cells [9], and outside the nervous system can be found in the stem cells of the gut [10] and epithelial cells of the mammary gland [11]. MSI2 is also present in differentiated neurons of the adult brain as well as in hematopoietic stem cells [12]. Expression of MSI1 has been correlated with the grade of malignancy and proliferative activity in gliomas and melanomas, whilst mutations in MSI2 have been associated with poor prognosis in certain types of cancers. Both MSI1 and MSI2 harbor two tandem RNA recognition motifs (RRMs) located at their N-terminal region, followed by a putative disordered region. The amino acid sequences of RRM1 and RRM2 of MSI1 exhibit 85% identity to those of MSI2 respectively, suggesting that the two proteins might target some mRNAs in a similar fashion. Indeed, an in vitro study identified the UAG RNA sequence as the core MSI1 binding motif [13], while recent genome-wide analyses of MSI2 targets revealed binding of MSI2 to mRNA 3’UTRs that contain multiple copies of UAG motifs as well as polyU pentamers [14, 15].

Dysregulated expression of RBPs can be detected in various human diseases ranging from neurological disorders to cancer [16, 17], rendering them potential targets for therapeutic intervention. However, identifying compounds that are able to disrupt protein-RNA interactions remains an arduous task due to the complexity of targeting RBPs or the selection of their interacting mRNAs. Previous studies have shed light on small molecule inhibitors of MSI1 in an effort to reduce Musashi-mediated tumourigenesis. Ω-9 fatty acids, such as oleic acid and its derivatives, were identified as allosteric inhibitors of MSI1 RNA-binding activity [18], while a fluorescence polarization (FP) competition assay revealed that (-)- gossypol, a natural compound extracted from cottonseed, acts as an in vitro inhibitor of MSI1 by recognizing and directly binding to its RNA-binding domain 1 (RBD1) [19].

*Caenorhabditis elegans* represents a powerful model organism to elucidate the molecular mechanisms of learning and memory. It is characterized by a simple nervous system of 302 neurons capable of exhibiting behavioral plasticity towards an array of stimuli [20, 21]. In *C. elegans,* the sole Musashi ortholog *(msi-1)* has been implicated in male mating [22]. We previously demonstrated that MSI-1 actively regulates forgetting via translational repression of actin branching complexes [7]. (-)- gossypol was the first small molecule compound to exhibit potent anticancer activity in vivo by disrupting MSI1 activity via the Notch/Wnt pathway [19], rendering it an ideal candidate to test for its potential effects on memory. In the current study, we investigated the evolutionary conserved role of MSI proteins and the effect of their pharmacological inhibition on memory and physiological age-dependent memory decline in these animals.

## Methods

### General Methods and Strains Used

Common reagents were obtained from Sigma (Sigma-Aldrich, St Louis, MO) unless otherwise indicated. Standard methods were used for maintaining and manipulating *C.* *elegans* [23]. All experiments were conducted using a synchronized population of young adult hermaphrodites. The *C.* *elegans* Bristol strain, variety N2, was used as the wild-type reference strain in all experiments. Alleles and transgenes used were the following: *msi-1(os1), msi-1(os1);utrIs15[p*_*msi-1*_*::human msi1cDNA::msi-1 3’UTR, p*_*sur-5*_*::mDsred], msi-1(os1);utrIs14[p*_*msi-*_*1::human msi2cDNA::msi1 3’UTR, p*_*sur-5*_*::mDsred], msi-1(os1);utrIs40[p*_*rig-3*_*::human msi1cDNA::msi-1 3’UTR, p*_*sur-5*_*::mDsred], msi-1 (utr55[YPET::3xFLAG]), utrSi43[prig-3::LoxP::BFP::LoxP::FLP-DS::SL2::GFP::H2B],unc-119(ed3), msi-1(utr55[YPET::3xFLAG]);utrSi43[prig-3::LoxP::BFP::LoxP::FLP-DS::SL2::GFP::H2B],unc-119(ed3), utrIs11[p*_*msi-1*_*::GFP::arx-2 3’UTR, punc-119* +*].*

Extrachromosomal transgenic lines were generated by injecting DNA at a concentration of 100 ng/μl into both arms of the syncytial gonad of worms as previously described (Mello et al., 1991). *psur-5::mDsRed* was used as a transformation marker at a concentration of 10 ng/μl. Chromosomal integration of extrachromosomal arrays was done by UV radiation for 12 s or 15 s. Following integration, generated strains were backcrossed four times to the wild-type strain.

### *C. elegans* Behavior Assays

Chemotaxis to different compounds was assessed in synchronized young adult worms as described previously [24]. Briefly, a population of well-fed, young adults was washed three times with CTX buffer (5 mM KH_2_PO_4_/K_2_HPO_4_ pH 6.0, 1 mM CaCl_2_, and 1 mM MgSO_4_) and 100–200 worms were placed in the center of a 10-cm CTX test plate. Worms were given a choice between a spot of attractant diluted in ethanol with 20 mM sodium-azide and a counter spot with ethanol and sodium-azide (NaN_3_). The distribution of the worms over the plate was determined after 1 h and the chemotaxis index (CI) was calculated as previously described (Chemotaxis Index = $\frac{\mathrm{no of worms in attractant }-\mathrm{no of worms incontrol}}{\mathrm{total amount of worms on plate}}$) [24]. Olfactory conditioning was assessed as previously described [25], with a few modifications. Starvation conditioning was performed without food in the presence of 2 μl undiluted diacetyl (DA) spotted on the plate for 1 h on 10 cm CTX plates (5 mM KH_2_PO_4_/K_2_HPO_4_ pH 6.0, 1 mM CaCl_2_, 1 mM MgSO_4_, 2% agar). Following conditioning, worms were tested for their chemotaxis towards DA immediately after or after 1 h rest to assess short-term associative memory.

Long-term associative memory was induced with two cycles of conditioning with DA with 30-min rest between trainings. Following conditioning, worms were kept on NGM plates in the presence of abundant food for 24 h and tested for chemotaxis to DA after the recovery phase [26].

Pharmacological treatment was performed by soaking worms for 2 h in CTX supplemented with either dimethyl sulfoxide (DMSO) alone or the indicated concentration of gossypol (AT101, Sigma-Aldrich) in DMSO, prior to olfactory conditioning. Gossypol was dissolved in absolute DMSO to make a 10 mM stock solution. After a 2-h incubation with the drug, worms were washed 3 times with CTX and tested for their preference to DA immediately (naïve), after conditioning, 1 h (STAM) or 24 h (LTAM) after conditioning. Furthermore, any potential delayed drug toxicity was tested 2 h and 24 h after treatment with the drug by assessing naïve chemotaxis to DA.

### Locomotory Rate Assays

Locomotory rate assays were carried out to test for any gossypol-related effects on worm motility. After treatment, worms were allowed to recover for 30 min on plates seeded with OP50. Individual worms were transferred on 6 cm assay plates, and allowed to move freely for 3 min and then the number of body bends of fifteen animals from each strain and treatment group as indicated was counted for 1 min.

### Molecular Biology

Rescue of the *msi-1(lf)* phenotype was performed with a 12.9-kb construct comprised of a 7.7 kb fragment of the *C. elegans msi-1* promoter region, the full-length *msi1* or *msi2* human cDNA designed with NcoI and KpnI compatible ends, fused with a 1.1 kb fragment of the *C. elegans msi-1* 3’UTR. Full-length human *msi1* and *msi2* cDNA were amplified from the MGC clones pENTR™223.1 and pOTB7 respectively (Transomic Technologies, Huntsville, AL, USA). Primers used for human *msi1* cDNA amplification were as follows: CCA TGG AGA CTG ACG CGC CCC AG and GGT ACC TCA GTG GTA CCC ATT GGT GAA GG and for human *msi2* cDNA amplification CCA TGG AGG CAA ATG GGA GCC AAG and GGT ACC TCA ATG GTA TCC ATT TGT AAA GG. The thermal cycle was programmed for 120 s at 95 °C as initial denaturation, followed by 30 cycles of 30 s at 95 °C for denaturation, 30 s at 64 °C as annealing, 90 s at 72 °C for extension, and final extension at 72 °C for 5 min. For the tissue-specific rescue experiments, the full-length *msi1* or *msi2* human cDNA and the *msi-1* 3’UTR were fused to a 3 kb fragment of the *rig-3* promoter using NEBuilder Hifi DNA assembly (New England Biolabs, Ipswich, MA).

### Reverse Transcription PCR

Total RNA was isolated from synchronized adult wild-type, *hmsi1* and *hmsi2* transgene-carrying animals using a Direct-zol RNA MiniPrep kit (Zymo Research Cooperation) with DNase treatment. RNA integrity was measured using the Agilent 2100 Bioanalyzer (Agilent Genomics, California, USA). cDNA was synthesized using SuperScript™ III First-Strand Synthesis System (ThermoFischer Scientific, MA, USA) according to the manufacturer’s recommendations using 1 µg of purified RNA. Primers designed to amplify human Musashi cDNA were the following: for *msi-1* ATT GAC CCT AAG GTG GCC TTC C and TGG CGG CGC TGA TGT AAC TG and for *msi-2* ACG TTC GCA GAC CCA GCA AG and TGG GAA GCC TGG GAA CTG ATA G. Plasmid DNA was used to test for primer annealing. To avoid amplification from contaminating genomic DNA, two sets of primers per construct were designed to span the endogenous *C. elegans msi1* promoter and human cDNA boundary region (*hmsi1*: ACG AAA CAA ACC ATG GAG AC and TGC GTA GTC TGC CAA CTG AG; CAA ACC ATG GAG ACT GAC GC and GAA GTA TTC GCG CAG CCC TT *hmsi2*: ACG AAA CAA ACC ATG GAG GCA and GGC TAT CTG GTG AGG TCT GC; CAA ACC ATG GAG GCA AAT GGG and CTA AGG CTA TCT GGT GAG GTC. Actin was used as a control for cDNA amplification with the following primers (forward primer: GCC CAA TCC AAG AGA GGT ATC C, reverse primer: GGC AAC ACG AAG CTC ATT G). Thermal cycle was programmed for 120 s at 95 °C as initial denaturation, followed by 30 cycles of 30 s at 95 °C for denaturation, 30 s at 60 °C as annealing, 60 s at 72 °C for extension, and final extension at 72 °C for 5 min.

### Targeted Modification of msi-1 Using CRISPR/Cas9

Endogenous tagging of *msi-1* with YPET::3xFlag was generated as described previously [27]. 601 bp and 722 bp homology arms flanking the N-terminus of *msi-1* were PCR amplified from N2 genomic DNA and inserted into the mNG^SEC&3xFlag vector pDD283 using NEBuilder Hifi DNA assembly (New England Biolabs, Ipswich, MA). The Cas9 target site was selected using the Sequence Scan for CRISPR database (http://cistrome.org/SSC/) and inserted into pDD162 [28]. The sgRNA sequence used was 5’- ATGACAACGACAGTATCAACGTTTTAGAGCTAGAAATAGCAAGT-3’. A mixture of 50 ng/µl Cas9–sgRNA plasmid, 10 ng/µl repair template, and 2.5 ng/µl pCFJ90, 5 ng/µl pCFJ104 and 10 ng/µl *sur-5p::dsRed* co-injection markers were injected into the gonads of young adults [29]. The knock-in line was established, the SEC cassette was excised using heat shock and the *msi-1* YPET::3xFlag line was sequenced to verify the correct insertion of the tag.

### Western Blot Analysis

For protein extraction, 50–100 worms per condition were lysed directly in ice-cold 3xLaemmli buffer (6%w/v SDS, 30%glycerol, 120 mM Tris–Cl pH 6.8, 0.03% w/v bromophenol blue) and heated for 5 min at 95 °C. Samples were directly subjected to SDS-PAGE, transferred to PVDF membranes, blocked with 5% nonfat dry milk in TBST (50 mM Tris–HCl, pH 7.5, 150 mM NaCl, 0.05% Tween-20) and incubated with primary antibodies as indicated. Antibodies used were mouse anti-FLAG (1:2000, Sigma Aldrich, St. Louis, MI) and mouse anti-tubulin (1:20,000, Merck Millipore, Burlington, MA). Primary antibodies were detected using HRP-coupled secondary antibodies (1:10,000, Jackson ImmunoResearch Laboratories, Cambridge House, UK). The chemiluminescent signal was developed using Clarity and ClarityMax Western Blotting Substrates (BioRad Laboratories Inc., Hercules, CA) followed by detection with a FujiFilm ImageQuant LAS-4000 detector (GE Healthcare, Chicago, IL).

### Fluorescence Microscopy

Whole worms were mounted on 3% agarose pads and immobilized with 0.5% NaN_3_. Synchronized 1-day-old adult worms were imaged using a Zeiss LSM 880 inverted scanning confocal microscope equipped with 25 × and 63 × oil immersion objectives. All recorded images were processed and quantified using Fiji software (version 2.1.0/1.53c). Fluorescence in AVA was measured using the integrated density function and the corresponding background signal was subtracted. Due to the dense neuronal signal in the head of the animal, AVA was first localized and selected in the BFP channel and the identical selection was then superimposed onto the YPET channel.

### Statistics

All data and statistical analyses were carried out using the Prism software (version: 9.1.2). Main effects and interaction terms were tested using ANOVA. Statistical tests for significance were done with F-tests using sum-of-squares type I. The *p*-value threshold was set to nominal significance (*p* < 0.05). In case of a significant main or interaction effect, the significance between data was tested using post hoc* t*-tests. *P*-values of the post hoc tests were corrected for the number of tests calculated per analysis (Bonferroni-correction per analysis: p_bonf_ < 0.05). For the imaging quantification, a two-tailed unpaired Student’s *t*-test was carried out to assess any differences between groups and the *p*-value threshold was set to nominal significance (*p* < 0.05). All figures were created using Adobe Illustrator (version 24.3.2).

## Results

### Human MSI1 and MSI2 Proteins Are Implicated in Memory

Previously, we have shown that the sole *C. elegans* Musashi ortholog, *msi-1*, actively promotes forgetting [7]. To study the potentially conserved role of MSIs in memory, we performed rescue experiments with the human Musashi homologues MSI1 (*Is[hsmi1* +*])* and MSI2 (*Is[hsmi2* +*])* in *C. elegans,* using established context-dependent associative learning and memory assays [25, 26].

**Fig. 1:**
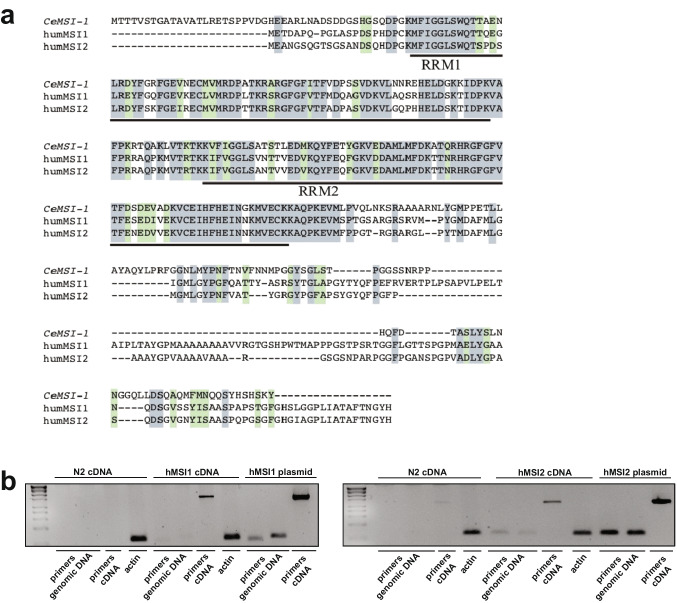
Sequence alignment of *C. elegans* and human Musashi orthologues and validation of transgene expression. (**a**) Grey boxes indicate identical amino acids and green boxes show similar amino acids. The two RNA-binding domains (RRM1 and RRM2), showing the highest conservation amongst species, are underlined. (**b**) Expression of human MSI1 and MSI2 was validated using cDNA extracted from the transgenic lines. N2 cDNA was used as a negative control for Musashi expression and the original plasmids used for cloning were used as positive controls for primer annealing. Actin was used as a further control for cDNA integrity. Primers annealing to the endogenous *msi-1* promoter/ human cDNA boundary identifies any amplification coming from contaminating genomic DNA (“primers genomic DNA”) and primers binding within the human cDNA region illustrate true expression (“primers cDNA”)

**Fig. 2:**
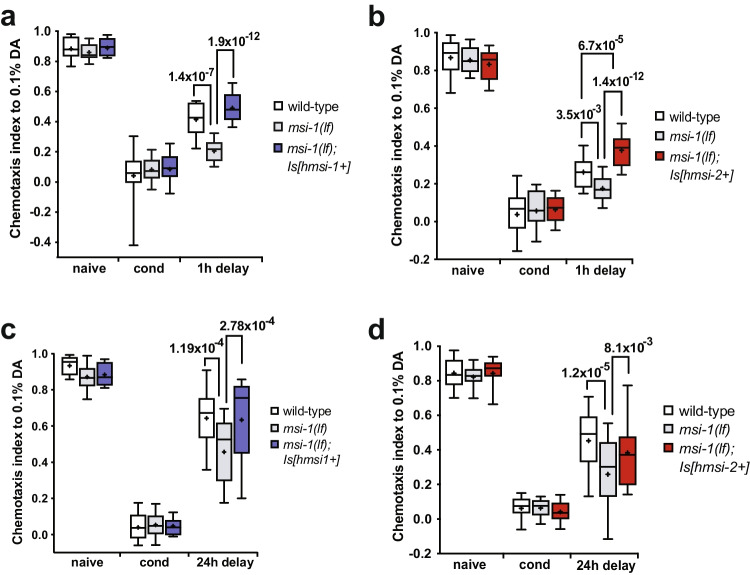
Human MSI1 and MSI2 are involved in the regulation of memory loss. (**a**, **b**) Negative STAM was tested in wild-type, *msi-1(lf)* and *msi-1(lf)* mutant worms rescued with the human *MSI1* or *MSI2* construct. Worms were assayed toward DA without (naïve), with preincubation with DA and starvation (cond) or after 1 h (1 h delay). Box plots for each genotype and condition are presented with whiskers indicating the 10th and 90th percentiles. Statistical significance between groups was assessed with two-way ANOVA (interaction effect for hMSI1: *F*_*(4, 153)*_ = *10.675, p* = *1.17* × *10*^*–7*^, interaction effect for hMSI2: *F*_*(4, 288)*_ = *10.9514, p* = *2.79* × *10*^*–8*^) and post hoc *t*-tests as indicated (Bonferroni’s adjusted *p* values are reported). (**c**, **d**) Negative LTAM in the different genotypes was tested following two consecutive conditioning phases and DA preference was tested immediately (cond) and after a 24 h (24 h delay) recovery period. Box plots for each genotype and condition are presented with whiskers indicating the 10th and 90th percentiles. Statistical significance between groups was assessed with two-way ANOVA (interaction effect for hMSI1: *F*_*(4, 153)*_ = *3.663, p* = *7.03* × *10*^*–3*^, interaction effect for hMSI2: *F*_*(4, 207)*_ = *3.63507, p* = *6.92* × *10*^*–3*^) and post hoc *t*-tests as indicated (Bonferroni's adjusted *p* values are reported). All experiments were done in triplicates and repeated at least five times

In order to study the functional conservation of Musashi proteins between worms and humans (Fig. 1a), we generated transgenic *C. elegans* lines expressing human MSI1 and MSI2 in a *msi-1* loss of function *(lf)* background. The *msi1(os1)* allele used in this study is a 1.6 kb deletion in the *msi-1* gene that results in a severely truncated protein that has no RRMs and is likely nonfunctional [22]. The transgenic animals were created by fusing 7.7 kb of the *C. elegans msi-1* promoter region to either the human *MSI1* or *MSI2* cDNA, followed by 1.1 kb of the *C. elegans msi-1* 3’UTR. Following integration of the extrachromosomal arrays, expression of both transgenes was confirmed with RT-PCR (Fig. 1b) and the lines were tested in a short- and long-term negative olfactory learning and memory paradigm. Initially, wild type, *msi-1(lf)*, *msi-1(lf); Is[hsmi1* +*]*, and *msi-1(lf); Is[hsmi2* +*]* animals were tested for their chemotaxis toward diacetyl (DA) prior to and following aversive associative learning (conditioning). All animals exhibited similar chemotaxis, independent of the genotype tested (Fig. 2a-d), suggesting that ectopic expression of human MSIs in worms does not influence baseline chemotaxis or associative learning. Moreover, we investigated the role of human MSI1 and MSI2 in short-term [STAM] and long-term [LTAM] associative memory. In the case of STAM, worms were subjected to one round of conditioning and tested for memory performance following a 1-h recovery period [25]. As previously shown, *msi-1(lf)* exhibited increased memory retention in short-term memory [7], a phenotype which was fully restored to wild-type levels in *msi-1(lf)* mutants carrying either the human *MSI1* or *MSI2* transgene (Fig. 2a-b). Similar to short-term memory, expression of either human *MSI*s fully rescued the effect of *msi-1(lf)* mutant worms on memory retention after a 24-h delay period (Fig. 2c-d) [7, 26]. Altogether, our results indicate that both human Musashi homologues are able to replace *C.elegans* MSI-1 protein function in STAM as well as in LTAM.

### Human MSI1 Protein Is Necessary in the AVA Interneuron

**Fig. 3:**
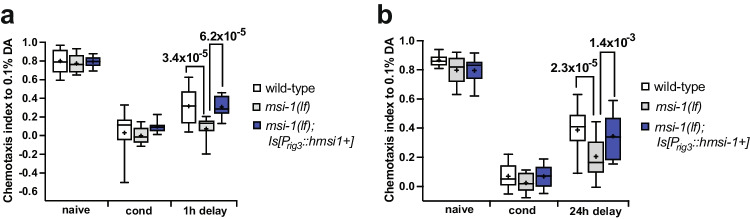
Human MSI1 regulates memory loss in the AVA interneuron. (**a**) Negative STAM was tested in wild-type, *msi-1(lf)* and *msi-1(lf)* mutant worms expressing the human *MSI1* construct. Worms were assayed toward DA without (naïve), with preincubation with DA and starvation (cond) or after 1 h (1 h delay). Box plots for each genotype and condition are presented with whiskers indicating the 10th and 90th percentiles. Statistical significance between groups was assessed with two-way ANOVA (interaction effect: *F*_*(4, 126)*_ = *3.423, p* = *1.07* × *10*^*–2*^) and post hoc *t*-tests as indicated (Bonferroni's adjusted *p* values are reported). (**b**) Negative LTAM in the different genotypes was tested following two consecutive conditioning phases and DA preference was tested immediately (cond) and after a 24 h (24 h delay) recovery period. Box plots for each genotype and condition are presented with whiskers indicating the 10th and 90th percentiles. Statistical significance between groups was assessed with two-way ANOVA (interaction effect: *F*_*(4, 153)*_ = *2.538, p* = *4.22* × *10*^*–2*^) and post hoc *t*-tests as indicated (Bonferroni's adjusted *p* values are reported). All experiments were done in triplicates and repeated at least five times

Previously, it was shown that expression of the *C. elegans* MSI-1 under the *rig-3* promoter, which drives expression primarily in the AVA interneuron, was sufficient to rescue the memory phenotype of *msi-1(lf)* mutants [7]. The AVA interneuron is located in the lateral ganglia of the head of the worm and is one of four bilaterally symmetric interneuron pairs (AVA, AVB, AVD, and PVC). It receives input from several upstream neurons (e.g., ASH, PVD, AFD) and sends out axons that run the entire length of the ventral nerve cord, providing input to neurons such as A-type (VA, DA) and AS motor neurons. AVA functions as a driver cell for backward locomotion (reversal) and was previously shown to play an important role in aversive long-term memory [7, 26]. To find out whether human MSI1 also shows a similar tissue-specific function as its *C.elegans* ortholog, we performed a rescue experiment by expressing the human *MSI1* cDNA specifically under the control of the *C. elegans rig-3* promoter in a *msi-1(lf)* background. As a result, we could observe rescue in both the STAM (Fig. 3a) as well as in the LTAM (Fig. 3b) testing paradigm suggesting that expression only in AVA is sufficient to restore the phenotype to wild-type.

### (-)- Gossypol Has No Adverse Effect on the Chemotactic or Locomotory Behavior of C. elegans

**Fig. 4:**
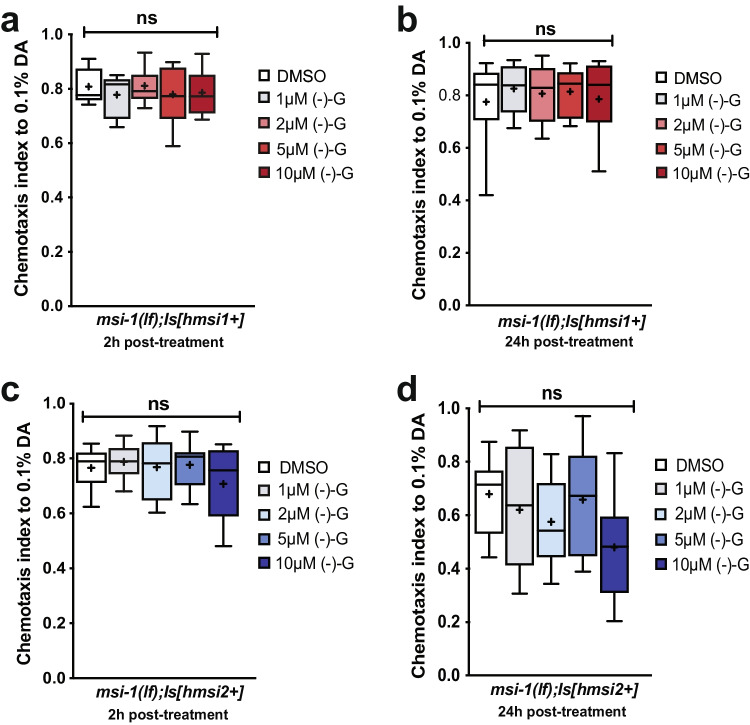
(-)- gossypol harbors no immediate or delayed toxicity. (**a**, **b**) Naïve chemotaxis was tested in *msi-1(lf)* mutant worms rescued with the human MSI1 construct following a (**a**) 2-h and (**b**) 24-h delay, post- (-)- gossypol treatment. Box plots for each genotype and condition are presented with whiskers indicating the 10th and 90th percentiles. Statistical significance between groups was assessed with one-way ANOVA (2 h delay effect: *F*_*(4, 70)*_ = *0.5650, p* = *0.6888,* 24 h delay effect: *F*_*(4, 55)*_ = *0.3248, p* = *0.86*) and post hoc *t*-tests as indicated (Bonferroni's adjusted *p* values are reported) ns, not significant. (**c**, **d**) Naïve chemotaxis was tested in *msi-1(lf)* mutant worms rescued with the human MSI2 construct following a (**c**) 2-h and (**d**) 24-h delay post- (-)- gossypol treatment. Box plots for each genotype and condition are presented with whiskers indicating the 10th and 90th percentiles. Statistical significance between groups was assessed with one-way ANOVA (2 h delay effect: *F*_*(4, 55)*_ = *1.190, p* = *0.3256,* 24 h delay effect: *F*_*(4, 55)*_ = *2.086, p* = *0.0951*) and post hoc *t*-tests as indicated (Bonferroni’s adjusted *p* values are reported) ns, not significant. All experiments were done in triplicates and repeated at least five times

Surface plasmon and nuclear magnetic resonance studies have demonstrated that (-)- gossypol directly interacts with the RNA-binding groove of MSI1 and disrupts binding to its downstream targets [19]. (-)- Gossypol’s therapeutic applicability has been limited due to the toxicity it harbors [30]. To address any potential toxic effects, we first measured the baseline chemotactic response of worms towards diacetyl following treatment with different (-)- gossypol concentrations (1–10 μΜ). In both human *MSI1* and *MSI2* transgenic worms, we observed no significant immediate (Fig. 4a-b) or delayed changes (Fig. 4c-d) in the chemotaxis index of worms treated with (-)- gossypol compared to untreated DMSO controls.

**Fig. 5:**
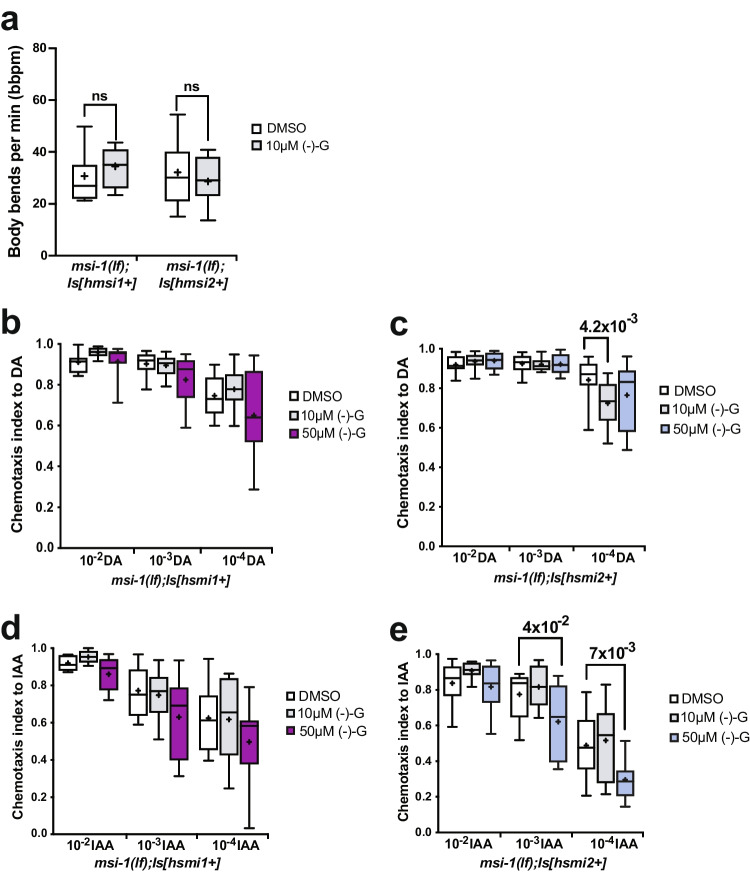
Effects of (-)- gossypol on the motoric and chemosensation behavior of worms. (**a**) Locomotory rate of DMSO and (-)- gossypol treated *msi-1(lf)* worms rescued with human MSI1 and human MSI2. Data were analyzed using a two-tailed Student’s test with *p* < 0.05. Box plots for each genotype and condition are presented with whiskers indicating the 10th and 90th percentiles. (**b**, **c**) Chemotaxis after (-)- gossypol treatment was measured towards DA as previously described [24] in *msi-1(lf)* worms rescued with human (**b**) MSI1 and (**c**) MSI2. Box plots for each genotype and condition are presented with whiskers indicating the 10th and 90th percentiles. Statistical significance between groups was assessed with two-way ANOVA (interaction effect for hMSI1: *F*_*(4, 99)*_ = *0.9442, p* = *0.44*, interaction effect for hMSI2: *F*_*(4, 99)*_ = *2.149, p* = *0.08*) and post hoc *t*-tests as indicated (Bonferroni’s adjusted p values are reported), ns, not significant. (**d**, **e**) Chemotaxis after (-)- gossypol treatment was measured towards IAA as previously described [24] in *msi-1(lf)* worms rescued with human (**d**) MSI1 and (**e**) MSI2. Box plots for each genotype and condition are presented with whiskers indicating the 10th and 90th percentiles. Statistical significance between groups was assessed with two-way ANOVA (interaction effect for hMSI1: *F*_*(4, 126)*_ = *0.2922, p* = *0.88*, interaction effect for hMSI2: *F*_*(4, 99)*_ = *1.181, p* = *0.32*) and post hoc *t*-tests as indicated (Bonferroni’s adjusted p values are reported), ns, not significant. All experiments were done in triplicates and repeated at least five times

To determine whether (-)- gossypol modulates the locomotory behavior of *C. elegans,* we measured the number of body bends per minute in worms treated with DMSO as vehicle control and 10 µM (-)- gossypol. Following a 2-h drug treatment, worms were transferred to clean OP50 seeded plates and allowed to move freely for 3 min before counting the locomotory rate. Using this measure, we found no difference in the locomotor response of *C. elegans* exposed to 10 µM (-)- gossypol versus the control group (Fig. 5a).

To investigate whether (-)- gossypol influences the chemosensation of worms, we measured the chemotactic response of DMSO-, 10 µM and 50 µM (-)- gossypol- treated worms towards different concentrations of diacetyl (DA) and isoamyl-alcohol (IAA). In *msi-1(lf)* worms rescued with the human *MSI1* construct, (-)- gossypol did not affect chemosensation at any given concentration of drug or chemoattractant (Fig. 5b, d). In *msi-1(lf)* worms rescued with the human MSI2 construct, we observed a reduction in the chemotaxis towards the lowest DA concentration (10^–4^) tested when worms were treated with 10 µM of (-)- gossypol (Fig. 5c). In contrast to DA, a high concentration of (-)- gossypol (50 µM) treatment significantly reduced the chemotactic response of *msi-1(os1);utrIs14[pmsi-1::human msi2, psur-5::dsred]* worms towards 10^–3^ and 10^–4^ IAA concentrations (Fig. 5e), suggesting that higher (-)- gossypol concentrations can impair the ability of these worms to detect low concentrations of odors. It is important to note that DA and IAA are detected via the AWA and AWC amphid sensory neurons respectively [24]; therefore, (-)- gossypol likely affects chemosensation independent of the sensory neuron implicated.

### (-)- Gossypol Inhibits Musashi-Mediated Forgetting and Improves Short- and Long-Term Memory

**Fig. 6:**
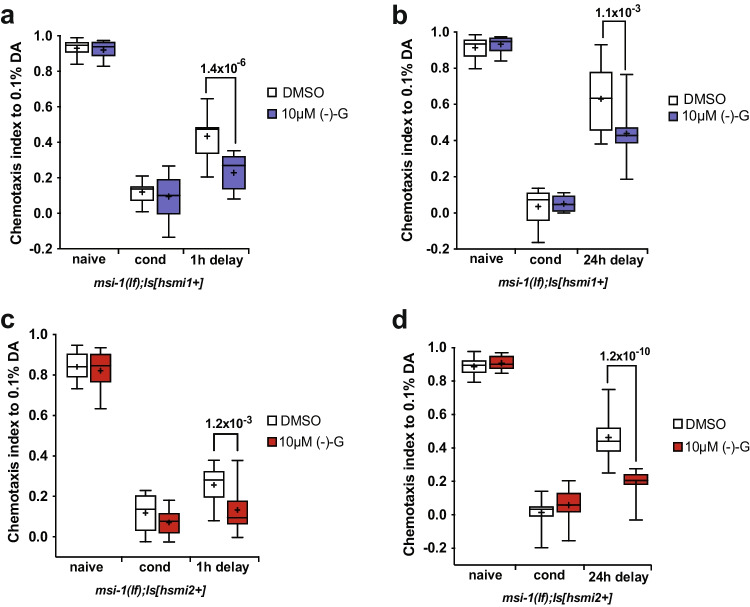
(-)- gossypol inhibits the memory rescue phenotype of human MSI1 and MSI2. (**a**, **b**) *msi-1(lf)* mutant worms rescued with the human MSI1 construct were tested for negative (**a**) STAM and (**b**) LTAM memory performance following a 2-h (-)- gossypol treatment. Worms were assayed toward DA without (naïve) or with preincubation with DA and starvation (cond) and after 1 h (1 h delay). Box plots for each genotype and condition are presented with whiskers indicating the 10th and 90th percentiles. Statistical significance between groups was assessed with two-way ANOVA (interaction effect for STAM: *F*_*(2, 84)*_ = *9.2769, p* = *2.28* × *10*^*–4*^, interaction effect for LTAM: *F*_*(2, 48)*_ = *5.18113, p* = *9.17* × *10*^*–3*^) and post hoc *t*-tests as indicated (Bonferroni’s adjusted *p* values are reported). (**c**, **d**) *msi-1(lf)* mutant worms rescued with the human MSI2 construct were tested for negative (**c**) STAM and (**d**) LTAM performance following a 2-h (-)- gossypol treatment. Negative LTAM in the different genotypes was tested following two consecutive conditioning phases and DA preference was tested immediately (cond) and after a 24 h (24 h delay) recovery period. Box plots for each genotype and condition are presented with whiskers indicating the 10th and 90th percentiles. Statistical significance between groups was assessed with two-way ANOVA (interaction effect for STAM: *F*_*(2, 100)*_ = *2.756, p* = *0.0684*, interaction effect for LTAM: *F*_*(2, 66)*_ = *19.7209, p* = *1.92* × *10*^*–7*^) and post hoc *t*-tests as indicated (Bonferroni’s adjusted *p* values are reported). All experiments were done in triplicates and repeated at least five times

Even though it is widely known that (-)- gossypol exhibits potent anti-tumor activity in vivo, no research has been done to shed light on its potential effects on memory. Thus, in order to pharmacologically modify MSI activity and memory, we investigated the possible effect of (-)- gossypol treatment on the memory performance of human MSI1- and MSI2-expressing *msi-1(lf)* animals. Treating worms with (-)- gossypol for 2 h prior to conditioning induced suppression of the phenotype observed in human MSI1-carrying worms compared to the DMSO treated line, thus improving memory in the STAM and LTAM behavioral assay (Fig. 6a-b). Similar findings were observed in human MSI2 expressing *C. elegans* lines, where (-)- gossypol inhibited the previously seen rescue of the forgetting defect phenotype, both in a STAM and LTAM behavioral assay (Fig. 6c-d). Thus, (-)- gossypol was able to improve both short- and long-term memory in either of the humanized *C.elegans* strains.

### (-)- Gossypol-Induced Memory Improvement Is Likely Mediated Through Neuron-Specific Musashi Inhibition

**Fig. 7:**
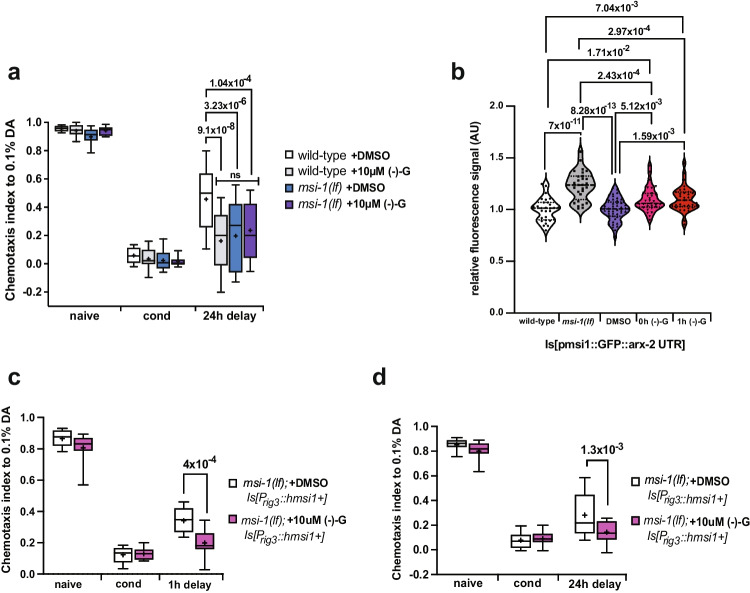
(-)- gossypol acts via the Musashi pathway to inhibit forgetting. (**a**) Negative LTAM was tested in wild-type and *msi-1(lf)* worms treated with DMSO and 10 µM (-)- gossypol. Box plots for each genotype and condition are presented with whiskers indicating the 10th and 90th percentiles. Statistical significance between groups was assessed with two-way ANOVA (interaction effect *F*_*(6, 204)*_ = *3.8519, p* = *1.15* × *10*^*–3*^) and post hoc *t*-tests as indicated (Bonferroni’s adjusted *p* values are reported). (**b**) GFP intensity in integrated transgenic worms expressing GFP under a 7.7-kb fragment of the *msi-1* promoter, fused to the *arx-2* 3′ UTR. GFP signal was measured in untreated worms expressing the array in a wild-type and *msi1 (lf)* background as well as in wild-type worms treated with either DMSO or (-)- gossypol (immediately or 1 h hour after treatment). Box plots for each genotype and condition are presented with whiskers indicating the 10th and 90th percentiles. Statistical significance between groups was assessed with one-way ANOVA (treatment effect *F*_*(4, 164)*_ = *20.51, p* = *1.01* × *10*^*–13*^) and post hoc *t*-tests as indicated (Bonferroni’s adjusted *p* values are reported). (**c**) Negative STAM was tested in *msi-1(lf)* mutant worms expressing human MSI1 under the *rig-3* promoter, treated with either DMSO and 10 µM (-)- gossypol. Worms were assayed toward DA without (naïve) or with preincubation with DA and starvation (cond) and after 1 h (1 h delay). Box plots for each treatment and condition are presented with whiskers indicating the 10th and 90th percentiles. Statistical significance between groups was assessed with two-way ANOVA (interaction effect *F*_*(2, 48)*_ = *4.756, p* = *0.0130*) and post hoc *t*-tests as indicated (Bonferroni’s adjusted *p* values are reported). (**d**) Negative LTAM was tested in *msi-1(lf)* mutant worms expressing human MSI1 under the *rig-3* promoter, treated with either DMSO or 10 µM (-)- gossypol. LTAM was tested following two consecutive conditioning phases and DA preference was tested immediately (cond) and after a 24 h (24 h delay) recovery period. Box plots for each treatment and condition are presented with whiskers indicating the 10th and 90th percentiles. Statistical significance between groups was assessed with two-way ANOVA (interaction effect *F*_*(2, 84)*_ = *4.040, p* = *0.0211*) and post hoc *t-*tests as indicated (Bonferroni’s adjusted *p* values are reported). All experiments were done in triplicates and repeated at least five times

It was previously shown that (-)- gossypol does not exclusively inhibit MSI1 activity, but can also act on other proteins, such as on the BCL-2 family members [31, 32]. To investigate the specificity of (-)- gossypol on MSI in the regulation of memory, we treated wild-type and *msi-1(lf)* worms with either DMSO or 10 µM (-)- gossypol and measured the effect on memory after a 24 h delay. While wild-type worms had a significant increase in LTAM retention when compared to their DMSO-treated control counterpart, *msi-1(lf)* worms displayed no differences between treatment groups (Fig. 7a).

Moreover, we investigated whether Musashi downstream targets change upon (-)- gossypol treatment. We focused on changes in the Arp2/3 actin member ACTR2/ARX-2, previously reported to interact with MSI-1 [7, 33] and whose abundance significantly increased in *msi-1 (lf)*worms [7] and upon (-)- gossypol treatment [33]. We treated worms expressing GFP under the control of the Musashi promoter, fused to the arx-2 3’UTR (pmsi-1::GFP::arx-2 3’UTR) with either DMSO or 10 µM (-)- gossypol for 2 h and quantified GFP levels of head neurons immediately or 1 h after treatment. We also measured GFP signals of the transgene in untreated wild-type and *msi-1 (lf)* worms as a control. In line with previous results, we observed a highly significant increase in the GFP signal in *msi-1 (lf)* worms compared to wild-type worms (Fig. 7b). DMSO-treated worms showed similar GFP expression levels to those of wild-type worms suggesting that DMSO treatment alone has no effect on ARX-2 protein levels (Fig. 7b). Wild-type worms treated with 10 µM (-)- gossypol showed a significant increase of GFP expression compared to untreated and DMSO-treated wild-type worms (Fig. 7b), suggesting that a 2-h treatment with (-)- gossypol is sufficient to partially inhibit Musashi and influence expression of its downstream targets.

The *msi-1* promoter has been suggested to drive expression in many tissues such as multiple neurons, muscle cells and the intestine (wormbase.org) which can have implications for the drug response. Consequently, we investigated whether (-)- gossypol has a tissue-specific mode of action rather than an off-target effect. We treated *msi-1(lf); p*_*rig-3*_*::human msi1cDNA::msi-1 3’UTR* worms with either DMSO or 10 µM (-)- gossypol and measured the memory phenotype after a 1-h or 24-h delay. In both the STAM (Fig. 7c) and LTAM (Fig. 7d) paradigms tested, (-)- gossypol treatment was sufficient to suppress the *msi-1* rescue phenotype previously shown in the AVA interneuron, further validating that the effect of (-)- gossypol on memory is unlikely driven by alternative, off-target pathways.

### (-)- Gossypol Treatment Does Not Influence MSI-1 Protein Abundance

**Fig. 8:**
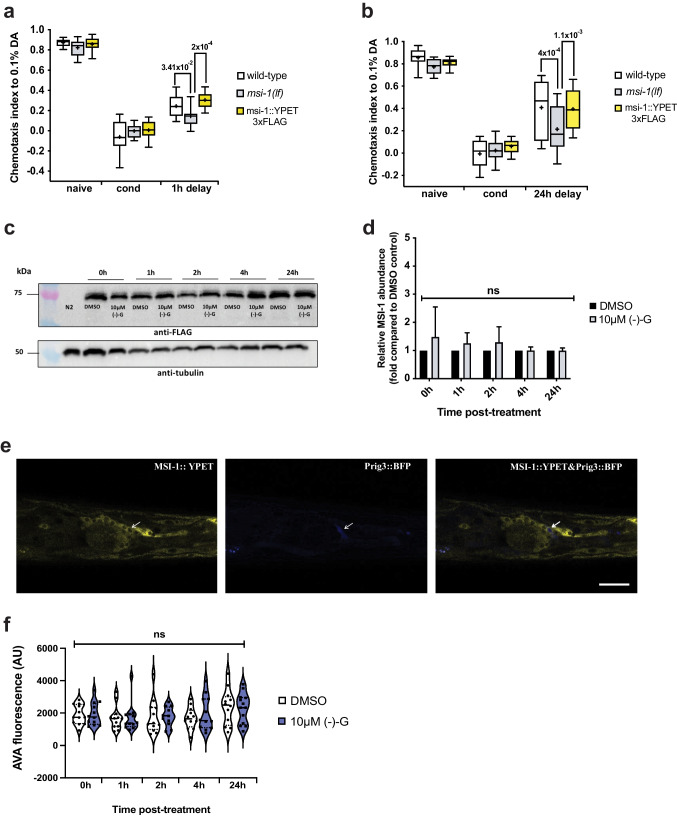
(-)- gossypol treatment does not influence MSI-1 protein abundance**.** (**a**) Negative STAM was tested in wild-type, *msi-1(lf)* and *msi-1::3xFLAG::YPET* worms. Worms were assayed toward DA without (naïve), with preincubation with DA and starvation (cond) or after 1 h (1 h delay). Box plots for each genotype and condition are presented with whiskers indicating the 10th and 90th percentiles. Statistical significance between groups was assessed with two-way ANOVA (interaction effect *F*_*(4, 125)*_ = *3.421, p* = *0.0109*) and post hoc *t*-tests as indicated (Bonferroni’s adjusted *p* values are reported). (**b**) Negative LTAM in the different genotypes was tested following two consecutive conditioning phases and DA preference was tested immediately (cond) and after a 24-h (24 h delay) recovery period. Box plots for each genotype and condition are presented with whiskers indicating the 10th and 90th percentiles. Statistical significance between groups was assessed with two-way ANOVA (interaction effect *F*_*(4, 153)*_ = *3.122, p* = *0.0167*) and post hoc *t*-tests as indicated (Bonferroni’s adjusted *p* values are reported). All experiments were done in triplicates and repeated at least five times. (**c**) MSI-1 protein abundance following DMSO or (-)- gossypol treatment was analyzed in a time-dependent manner using western blots. For each condition, 50–100 transgenic worms were analyzed using FLAG antibody (top) and as the loading control membranes were reprobed for tubulin (bottom). (**d**) Relative MSI-1 abundance is plotted at different time points following a 2 h (-)- gossypol treatment and values are presented as a fold change to the DMSO control group. Bars represent mean ± SD from 6 independent blots. Statistical significance between groups was assessed with two-way ANOVA (interaction effect *F*_*(4, 50)*_ = *0.8197, p* = *0.5187*), ns, not significant. (**e**) Representative confocal images of MSI-1 expression in the AVA interneuron (arrows) in MSI-1::YPET::3xFLAG animals. Scale bar, 20 μm. (**f**) Violin plots displaying quantification of fluorescence intensity in AVA interneuron in MSI-1::YPET::3xFLAG animals at different time points following DMSO and (-)- gossypol treatment. Animals were recorded with identical microscope settings and YPET intensity was measured on z-projected confocal images and quantified using ImageJ software. The center line of the violin plot represents the group median, whereas the bottom and top lines represent the 25^th^ and 75^th^ percentiles respectively. Statistical analysis was performed using a two-way ANOVA (interaction effect *F*_*(4, 111)*_ = *0.1538, p* = *0.9609),* ns, not significant

To investigate whether any of the effects of (-)- gossypol could be a result of altered protein expression rather than a result of inhibition of protein function, we conducted a time course assay and examined MSI-1 endogenous protein levels after treatment. We first tagged the endogenous gene at the N-terminal end with a yellow fluorescent protein (YPET) and 3xFLAG using CRISPR/Cas9 (Dickinson et al., 2015). To confirm the functional integrity of the tagged protein, we compared the memory performance between wild-type, *msi-1(lf)* and MSI-1::YPET::3xFLAG animals and found no difference in either STAM or LTAM (Fig. 8a-b).

To check for possible (-)- gossypol-related changes in MSI-1 protein abundance, we treated worms for 2 h with either DMSO or 10 μM gossypol and collected worm fractions at different time points. By carrying out western blot analysis, we could not detect any significant changes in MSI-1 protein levels (Fig. 8c-d), suggesting that (-)- gossypol inhibits MSI-1 function without affecting its abundance. Since the fractions collected are whole worm lysates and represent MSI-1 global expression, we carried out fluorescent microscopy to identify any tissue-specific MSI-1 expression changes. We focused specifically on the AVA neuron and crossed the MSI-1::YPET::3xFLAG strain with an integrated *rig-3* promoter-driven BFP line to easily localize the AVA interneuron amongst the dense head neuronal network (Fig. 8e). We could not detect any significant changes in MSI-1 expression at different time points following treatment, further corroborating our western blot results (Fig. 8f).

### Effect of msi-1 and (-)- Gossypol on Age-Dependent Cognitive Decline

**Fig. 9:**
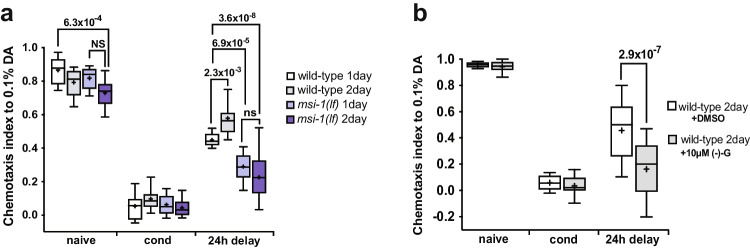
(-)- Gossypol reverses physiological age-dependent cognitive decline in a MSI-1-dependent manner**.** (**a**) Negative LTAM was tested in 1-day and 2-day-old wild-type and *msi-1(lf)* worms. Worms were assayed toward DA without (naïve), with two rounds of preincubation with DA and starvation (cond) or after 24 h (24 h delay). Box plots for each genotype and condition are presented with whiskers indicating the 10th and 90th percentiles. Statistical significance between groups was assessed with two-way ANOVA (interaction effect *F*_*(6, 129)*_ = *11.901 p* = *1.33* × *10*^*–10*^*)* and post hoc *t*-tests as indicated (Bonferroni’s adjusted *p* values are reported). (**b**) Negative LTAM was assessed in 2-day-old wild-type worms treated with DMSO and 10 µM (-)- gossypol. Box plots for each genotype and condition are presented with whiskers indicating the 10th and 90th percentiles. Statistical significance between groups was assessed with two-way ANOVA (interaction effect *F*_*(2, 66)*_ = *14.9156 p* = *4.52* × *10*^*–6*^*)* and post hoc *t*-tests as indicated (Bonferroni’s adjusted *p* values are reported). All experiments were done in triplicates and repeated at least five times

Physiological, age-dependent memory decline can be readily observed in nematodes with 2-day-old worms already exhibiting a significant decline in olfactory associative memory [34]. To investigate the potential beneficial effect of *msi-1* inhibition on age-dependent cognitive decline, we compared LTAM in 1- and 2-day-old wild-type and *msi-1 (lf)* worms. As expected, the LTAM of wild-type worms significantly decreased with age, whilst *msi-1 (lf)* displayed no age-dependent memory decline (Fig. 9a). Furthermore, both 1- and 2-day-old *msi-1 (lf)* worms exhibited a significant increase in LTAM retention compared to their wild-type counterparts. In line with previous results, (-)- gossypol treatment of 2-day-old wild-type worms mimicked the *msi-1 (lf)* phenotype, with animals displaying improved LTAM retention when compared to their DMSO-treated counterparts (Fig. 9b), suggesting that (-)- gossypol could help improve memory of aged worms.

## Discussion

A nervous system’s robust cognitive fitness does not stem solely from its ability to form new memories but also from its capacity to erase/forget irrelevant and harmful information [5]. Forgetting should not be regarded as having a negative connotation, but rather be seen as a dynamic cognitive process of the brain, enabling us to adapt and survive in a constantly changing environment. Thus, forgetting constitutes an innate system characterized by its own finely tuned molecular and cellular processes that should be distinguished from those regulating memory encoding, consolidation and retrieval [5].

Here we report that the human Musashi orthologues, MSI1 and MSI2, likely have a conserved role in the regulation of memory. Introducing human MSIs into *C. elegans* under the control of either the endogenous *msi-1* or *rig-3* promoter efficiently rescued the forgetting defect of *msi-1(lf)* worms, both in a short-term and long-term aversive associative learning paradigm. This suggests that despite the differences in the amino acid sequence between worm and human MSI proteins, their function seems to be conserved during the regulation of forgetting. This is the first study to suggest that human MSI proteins could have a role in the active regulation of forgetting. In addition, pharmacological treatment of human MSI1- and MSI2-expressing worms with the natural compound (-)- gossypol, improved memory with no effects on locomotion or memory acquisition of worms. MSI1 and MSI2 display a high degree of sequence and structure similarity and appear to target the same mRNA recognition motif [35]. Therefore, (-)- gossypol could be considered as an MSI1/2 dual inhibitor. 

The (-)- gossypol enantiomer used in this study is eliminated slowly [36, 37], constitutes the most biologically active form of the compound and consequently is considered more toxic than (+) gossypol [38]. Toxicity testing assays revealed no immediate or delayed toxic effects of (-)- gossypol concentrations up to 10 µM, however, higher concentrations (50μΜ) exhibited slight toxic effects, as illustrated by the reduced chemotactic response of worms (Fig. 5). Additionally, (-)- gossypol did not alter the locomotory behavior of worms, which could influence their response towards the chemoattractant diacetyl. However, in mammals, high concentrations of free gossypol have been shown to cause respiratory distress, impaired body weight gain, weakness, apathy, impaired reproduction, compromised immune function and even death [30]. Thus, gossypol toxicity constitutes a strong limitation when it comes to using it as a pharmacological inhibitor for treating forgetting in humans. However, a recent study by Lan et al., 2018 revealed gossypolone (Gn), a (-)- gossypol metabolite, as a 20-fold more potent disruptor of MSI activity [39]. This could address the issue of toxicity discussed previously, since much lower concentrations would be required to achieve the same effect. Additionally, it should be pointed out, that the lack of toxicity in *C. elegans* does not preclude it in humans. Previous studies have shown how worm pharmacology can differ, where dopamine drugs had opposing effects on DA receptors in worms and mammals [40] and sodium azide is a permanent cytochrome c oxidase inhibitor in mammals whilst a reversible one in *C. elegans*. Thus, we acknowledge that caution must be paid for when using *C. elegans* as a model for drug screening.

Furthermore, (-)- gossypol has been shown not to be selective against MSI but can act on multiple pathways [41, 42]. However, we did not observe any behavioral changes in (-)- gossypol-treated *msi-1(lf)* worms. Moreover, (-)- gossypol treatment of worms expressing human MSI1 solely in the AVA interneuron inhibits the previously seen phenotypic rescue (Fig. 3), suggesting a tissue-specific mode of drug action rather than an off-target effect. Nevertheless, while the concentrations used in our treatment might not suffice to affect other pathways, we cannot exclude that (-)- gossypol could harbor off-target effects, as increased toxicity is observed with increasing concentrations of the drug. Future efforts should, therefore, focus on elucidating compounds that selectively target and inhibit MSI1/2.

Finally, physiological age-dependent cognitive decline normally observed in wild-type worms did not occur in 2-day-old *msi-1(lf)* worms or 2-day-old wild-type worms treated with (-)- gossypol. These results implicate Musashi in age-dependent memory decline and nominate Musashi-inhibiting compounds as memory modulators.

Taken together, our results argue in favor of an evolutionary conserved role for Musashi proteins in memory in species as diverse as nematodes and humans. Additionally, we show that *C. elegans*, similar to other model organisms [43], can be successfully used as a model for therapeutic intervention using pharmacological manipulations [44], which might prove useful in the treatment of memory-related disorders.

## Data Availability

Not applicable.
